# Viral Hepatitis Surveillance — India, 2011–2013

**DOI:** 10.15585/mmwr.mm6428a3

**Published:** 2015-07-24

**Authors:** Tripurari Kumar, Aakash Shrivastava, Anil Kumar, Kayla F. Laserson, Jai P. Narain, Srinivasaraghavan Venkatesh, Lakhbir S. Chauhan, Francisco Averhoff

**Affiliations:** 1 India Epidemic Intelligence Service Program, National Centre for Disease Control (NCDC); 2 Epidemiology Division, NCDC; 3 Division of Global Disease Detection, Center for Global Health CDC India; 4 Integrated Disease Surveillance Program, NCDC; 5 Office of the Director, NCDC; 6 Division of Viral Hepatitis, National Center for HIV/AIDS, Viral Hepatitis, STD, and TB Prevention, CDC

The burden of viral hepatitis in India is not well characterized. In 2009, the national Integrated Disease Surveillance Programme (IDSP) began conducting surveillance across all Indian states for epidemic-prone diseases, including foodborne and waterborne forms of viral hepatitis (e.g., hepatitis A and E). Information on outbreaks of all forms of viral hepatitis, including A, B, C, and E, also is collected. This report summarizes viral hepatitis surveillance and outbreak data reported to IDSP during 2011–2013. During this period, 804,782 hepatitis cases and 291 outbreaks were reported; the virus type was unspecified in 92% of cases. Among 599,605 cases tested for hepatitis A, 44,663 (7.4%) were positive, and among 187,040 tested for hepatitis E, 19,508 (10.4%) were positive. At least one hepatitis outbreak report was received from 23 (66%) of 35 Indian states. Two-thirds of outbreaks were reported from rural areas. Among 163 (56%) outbreaks with known etiology, 78 (48%) were caused by hepatitis E, 54 (33%) by hepatitis A, 19 (12%) by both hepatitis A and E, and 12 (7%) by hepatitis B or hepatitis C. Contaminated drinking water was the source of most outbreaks. Improvements in water quality and sanitation as well as inclusion of hepatitis A vaccine in childhood immunization programs should be considered to reduce the public health burden of hepatitis in India. Efforts to decrease the proportion of cases for which the etiology is unspecified, including expanding the IDSP to support hepatitis B and C testing, might help further elucidate the epidemiology of these diseases.

India is known to have a large burden of viral hepatitis (1–4), but national surveillance data are lacking. In 2009, IDSP, operated through India’s National Center for Disease Control (NCDC), became active in all Indian states (5). Weekly surveillance data on 18 epidemic-prone diseases, including viral hepatitis, are collected through this program. All 28,850 government-run primary health care centers and hospitals and 2,923 designated private facilities serve as reporting units, which collect and report data on hepatitis cases (any acute onset of jaundice) and outbreaks, and report them to district surveillance units each week. These reports are submitted as aggregate data to IDSP through a web portal ( http://www.idsp.nic.in ); no demographic information, risk factors, or other data are collected or reported.

The district surveillance units also investigate suspected hepatitis outbreaks (two or more epidemiologically linked cases of acute jaundice). IDSP supports testing for hepatitis A and E, and during outbreaks, testing for hepatitis B and C also is supported. Outbreak investigation reports include a description of the affected population, number of cases and deaths, date of onset of the first case, laboratory data, information on the suspected source of the outbreak, and control measures undertaken. Hepatitis outbreaks are classified by etiology when at least one case is laboratory-confirmed and the others are epidemiologically linked. Cases are categorized as hepatitis A, B, C, E, or unspecified if the etiology is not determined. NCDC operates a national outbreak-monitoring call center and a national media scanning center to identify suspected outbreaks and, after investigation, also compiles them into weekly national alerts. This report summarizes an analysis of 2011–2013 national viral hepatitis surveillance and outbreak data from IDSP and weekly national alerts. Census data from 2011 were used to calculate incidence.

During 2011–2013, a total of 804,782 viral hepatitis cases were reported to IDSP. Among 599,605 (74.5%) cases tested for hepatitis A, 44,663 (7.4%) were positive, and among 187,040 (23.2%) tested for hepatitis E, 19,508 (10.4%) were positive. The etiology of 740,611 (92%) reported cases was not determined (Figure 1 and Figure 2). During June–September of each reporting year, a 17% increase in the total number of reported hepatitis cases above baseline was observed, and laboratory-confirmed hepatitis A cases followed the same seasonal pattern with an average increase of 18% (Figure 2). During the 3-year period, eight states had average annual rates of >50/100,000 total hepatitis cases, whereas no state reported rates of ≥10/100,000 hepatitis A or E cases during any year of the reporting period.

During the 3-year period, 291 hepatitis outbreaks involving 15,601 cases and 58 (4%) deaths were reported to IDSP. Outbreak-related cases accounted for 1.9% of all reported hepatitis cases. Twenty-three (65.7%) of India’s 35 states reported at least one hepatitis outbreak; five states reported >20 outbreaks (Figure 3). More outbreaks were reported from rural areas (199 [68%]) than urban areas (92 [32%]); 163 (56%) outbreaks were laboratory-confirmed, and, of those, most were either hepatitis E (78 [47.9%]) or hepatitis A (54 [33.1%]). Additionally, both hepatitis A and E were reported in 19 outbreaks, and hepatitis B or C, or both, was reported as the etiology of 12 outbreaks. Contaminated drinking water was identified as a cause for 72% (109 of 151) of the hepatitis A and E outbreaks, and was implicated in 49 (38%) of the 128 outbreaks for which laboratory confirmation was not available.

## Discussion

This is the first report of national viral hepatitis surveillance and outbreak data from India. Although a specific etiology was not confirmed for most reported cases, hepatitis cases and outbreaks caused by hepatitis A and E were regularly reported from most regions, and a seasonal variation in hepatitis A cases was recognized, although no seasonal pattern was observed for outbreaks. Consistent with previous reports from India (1,2), unsafe drinking water was the most commonly reported cause of hepatitis A and E outbreaks, highlighting the need for improved access to clean drinking water and improved sanitation. Although IDSP does not routinely support laboratory testing for hepatitis B and C, it does support testing during outbreaks, resulting in some hepatitis B and C outbreaks being detected. This finding suggests a potential benefit of including hepatitis B and C testing of nonoutbreak cases reported to IDSP to better understand the burden and epidemiology of these pathogens. The small proportion of jaundice cases tested for either hepatitis A or E that tested positive, 7% and 10%, respectively, needs further investigation. The low number of laboratory-confirmed cases could be the result of misclassification of clinical cases, laboratory error, delays in testing, or large numbers of acute hepatitis that are neither A nor E. Some states with the highest reported number of outbreaks were among those supported by the World Bank for surveillance infrastructure strengthening (6), and better surveillance in these states might account for the increased number of cases as well as outbreaks reported, rather than an actual greater number of outbreaks in these states.

Surveillance for hepatitis often underestimates the actual number of cases. Nevertheless, IDSP identified a substantial number of hepatitis cases and outbreaks during 2011–2013. The large number of hepatitis A and E outbreaks might be explained in part by the lack of adequate sewage and sanitation systems (1); defecation in open fields, which can contaminate surface drinking-water sources, remains a common practice. The large numbers of hepatitis A cases might also reflect an epidemiologic shift in the affected population in India. Hepatitis A infection during childhood often is asymptomatic and unrecognized, and typically confers lifelong immunity. With increasing age at time of infection, symptomatic cases become more common. With improved hygiene and sanitation reflecting India’s improving economy, more children might escape childhood infection and remain susceptible to infection during adolescence and adulthood (7). Demographic data, including age, not currently included in IDSP, would help to better understand the epidemiology of hepatitis A in India. Such data also could be used to inform consideration of inclusion of hepatitis A vaccine in the routine immunization program.

Peaks in reporting occurred during the monsoon season (June–September) for both total cases reported and hepatitis A cases reported during each of the reporting years. This pattern suggests that most unspecified cases might be hepatitis A, and that there is seasonal variation in transmission of hepatitis A, possibly related to contamination of drinking water during periods of heavy rain.

Hepatitis B and C cause substantial morbidity and mortality worldwide. Although poorly described in India, hepatitis B and C are thought to contribute substantially to the country’s overall hepatitis burden (3,4). Through IDSP, laboratory support has been steadily strengthened, and most states have at least one public health laboratory (5,6); however, routine laboratory testing of suspected hepatitis cases for hepatitis B and C is not currently supported by IDSP. Inclusion of such testing would improve understanding of the epidemiology of hepatitis B and C and relevant risk factors. Further, surveillance for chronic hepatitis, cirrhosis, and hepatocellular carcinoma would give valuable insights into the long-term disease burden in the country (2).

The findings in this report are subject to at least four limitations. First, the finding that more hepatitis outbreaks are reported from rural than urban areas might partially be explained by greater government-sponsored health care delivery in rural areas, which might be more likely to identify and report outbreaks to IDSP. Second, the majority of reported cases were not laboratory-confirmed. Third, data were available for only a few hepatitis B and C outbreaks, limiting the use of data from those investigations. Finally, incomplete follow-up and reporting of outbreaks to IDSP might lead to an underestimation of the burden and an inadequate understanding of the epidemiology of the outbreaks.

Routine disease surveillance is a core public health function. The formation of IDSP is a major advance toward building India’s public health capacity to identify and react to urgent threats and monitor disease trends. Hepatitis surveillance data obtained through IDSP can be used to monitor disease trends, identify local hepatitis outbreaks, and to evaluate the effectiveness of sanitation, safe water, immunization, and other prevention and control measures. To enhance the utility of its data, IDSP might consider introducing case-based surveillance that includes demographic and risk factor data, improving geographic representativeness of surveillance data, and increasing the proportion of cases that are laboratory-confirmed. Further, increasing laboratory capacity to include hepatitis B and C into routine testing might help identify unrecognized modes of transmission and populations at risk for infection (4).

**Summary**

What is already known on this topic?

Hepatitis A and hepatitis E are endemic in India, and although hepatitis B and hepatitis C are thought to be common, national data are lacking on all forms of viral hepatitis.

What is added by this report?

The National Integrated Disease Surveillance Program, established in India in 2009, collects data on cases and outbreaks of jaundice, and supports outbreak investigations and laboratory testing for hepatitis A and hepatitis E. During 2011–2013, large numbers of hepatitis A and hepatitis E cases and frequent outbreaks occurred each year. Hepatitis A and hepatitis E outbreaks were reported throughout the country, associated with poor water quality and lack of sanitation. Cases of hepatitis A appeared to follow a seasonal pattern associated with the monsoon season.

What are the implications for public health practice?

Epidemiologic and laboratory strengthening of the Integrated Disease Surveillance Program might improve understanding of the hepatitis disease burden in India because most cases were not laboratory-confirmed. Further, the large numbers of cases and outbreaks underscore the need for improvements in water quality and sanitation. Finally, collection of additional demographic and epidemiologic data on hepatitis A can inform consideration of including hepatitis A vaccine in routine immunization programs.

## Figures and Tables

**FIGURE 1:**
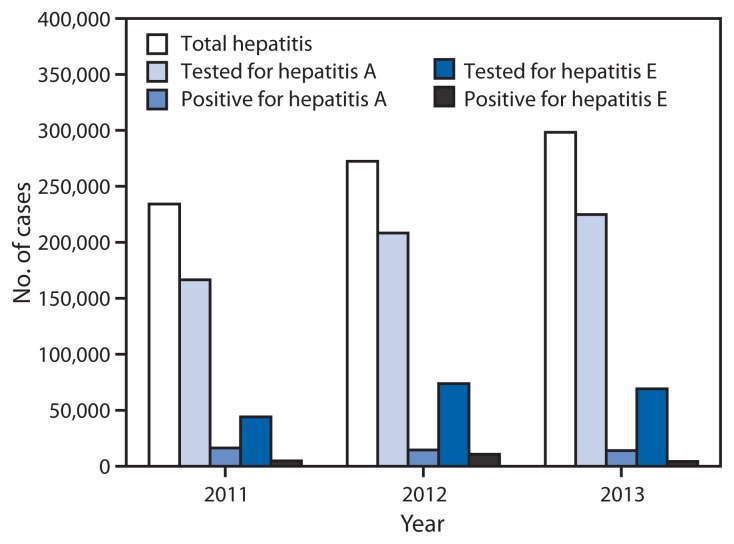
Number of hepatitis cases reported, number tested, and number confirmed for hepatitis A and hepatitis E* — India, 2011–2013 * Nos. of cases tested for hepatitis A and E are not mutually exclusive.

**FIGURE 2:**
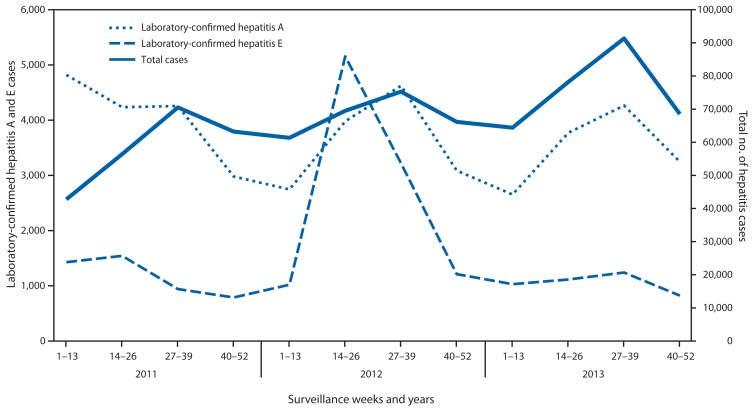
Total number of hepatitis cases reported and number laboratory-confirmed as hepatitis A and hepatitis E, by surveillance weeks — India, 2011–2013

**FIGURE 3:**
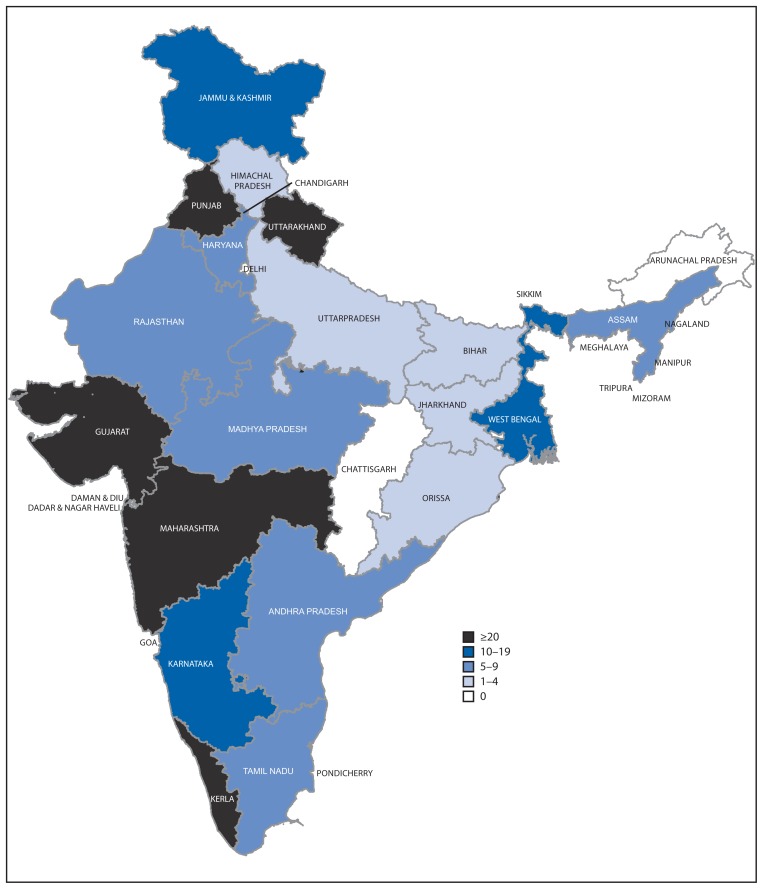
Number of reported hepatitis outbreaks (N = 291), by state — India, 2011–2013

## References

[1] Acharya SK (2013). This is hepatitis: know it, confront it. Indian J Med Res.

[2] John TJ, Dandona L, Sharma VP, Kakkar M (2011). Continuing challenge of infectious diseases in India. Lancet.

[3] Batham A, Gupta MA, Rastogi P, Garg S, Sreenivas V, Puliyel JM (2009). Calculating prevalence of hepatitis B in India: using population weights to look for publication bias in conventional meta-analysis. Indian J Pediatr.

[4] Mukhopadhyaya A (2008). Hepatitis C in India. J Biosci.

[5] Suresh K (2008). Integrated Diseases Surveillance Project (IDSP) through a consultant’s lens. Indian J Public Health.

[6] The World Bank (2012). Report no: ICR2203. Implementation completion and results report.

[7] Mathur P, Arora NK (2008). Epidemiological transition of hepatitis A in India: issues for vaccination in developing countries. Indian J Med Res.

